# Hydrological hazards in Vhembe district in Limpopo Province, South Africa

**DOI:** 10.4102/jamba.v11i2.698

**Published:** 2019-06-25

**Authors:** John Odiyo, Fhumulani I. Mathivha, Tinyiko R. Nkuna, Rachel Makungo

**Affiliations:** 1 Department of Hydrology and Water Resources, School of Environmental Sciences, University of Venda, Thohoyandou, South Africa

**Keywords:** Climatic Variability and Changes, Consequence Level, Drought and Flood Risk Rating, Hydrological Hazards, Mitigation Strategies, Risk and Vulnerability

## Abstract

This study determined the risks associated with hydrological hazards and vulnerabilities to communities in Vhembe District Municipality, Limpopo province. Risk and vulnerability contribute to poverty, loss of lives and property, environmental and infrastructural destruction, food insecurity and unavailability of water resources. Streamflow and rainfall data were analysed using Log-Pearson Type III distribution and Standardised Precipitation Index (SPI), respectively, to identify return periods and probabilities of occurrence of floods and droughts. Mann–Kendall test was applied to identify trends of floods and droughts. Risk ratings were used to determine risks and vulnerabilities associated with floods and droughts. Standardised Precipitation Index analysis showed that a mild dryness condition dominated dry years in all stations with a range of 22.4% to 59.2% of the years falling within this category. Twenty-five per cent and 75% of rainfall stations depicted downward and upward trends, respectively. Equal number of streamflow stations depicted downward and upward trends. Results generally showed that flood events with return periods of 50, 100 and 200 years are mostly associated with significant and catastrophic consequence levels. This demonstrated high risk and vulnerability of the communities to these hazards. The findings of this study will aid in future planning and development of mitigation strategies associated with hydrological hazards.

## Introduction

Hydrological hazards are hydrological events capable of inflicting damage to human and animal life and/or property (Mulugeta et al. 2007). Spatio-temporal variability in precipitation is one of the factors that increase the occurrence and risks of these hazards. The most common hydrological hazards in southern Africa are floods and droughts because of extreme weather patterns associated with a highly variable climate. Convective clusters associated with the Inter-Tropical Convergence Zone (ITCZ) are believed to produce very strong gusty winds and torrential rainfall, which result in floods in southern Africa (Moolchan 2010). El Niño–Southern Oscillation’s influence also gives rise to floods and droughts, manifested in La Nina and El Nino, respectively. Droughts and floods have also become common because of increases in temperatures. In Limpopo province, temperatures have risen by 1°C over recent years (Gbetibuou 2009). Drought is the usual aftermath of southern Africa’s climatic changes and is one of the most important natural disasters in southern Africa (Unganai 1994). Throughout the 20th century, droughts have occurred over South Africa with great regularity (Vogel 1995). The 1991–1992 period witnessed one of the worst recent droughts recorded in the country because of far-reaching impacts felt through all sectors of society (Glantz, Betsill & Crandall 1997; Vogel, Laing & Monnik 2000).

Merz, Elmer and Thieken (2009) defined risk as the combination of the probability of a particular event and the impact that the event would cause. Majority of the risk reduction research and practice in South Africa is orientated towards structural measures or technical/hydrological component of the non-structural early warning systems (Benjamin 2008). Studies that integrate assessment of hydrological hazards, their trends, associated risks and vulnerabilities in South Africa are lacking. Vhembe District Municipality (VDM) (2011) listed floods and droughts as major disasters that occur in the study area. Given the variability in rainfall combined with poor infrastructure in some parts of VDM, the level of vulnerability increases tremendously. Disaster management by VDM is limited to taking post-disaster measures and implementing few early warning systems (Musyoki, Thifhufhelwi & Murungweni 2016). Thus, identification of hazards (including their trends, probabilities of occurrence and associated risks and vulnerabilities), as has been undertaken in this study, is important. This will support disaster risk management in the study area. Flood frequency analysis (FFA) is also a part of hazard identification and risk assessment. Thus, the findings of this study will aid in strategic mitigation of hydrological hazards, in the future.

**FIGURE 1:**
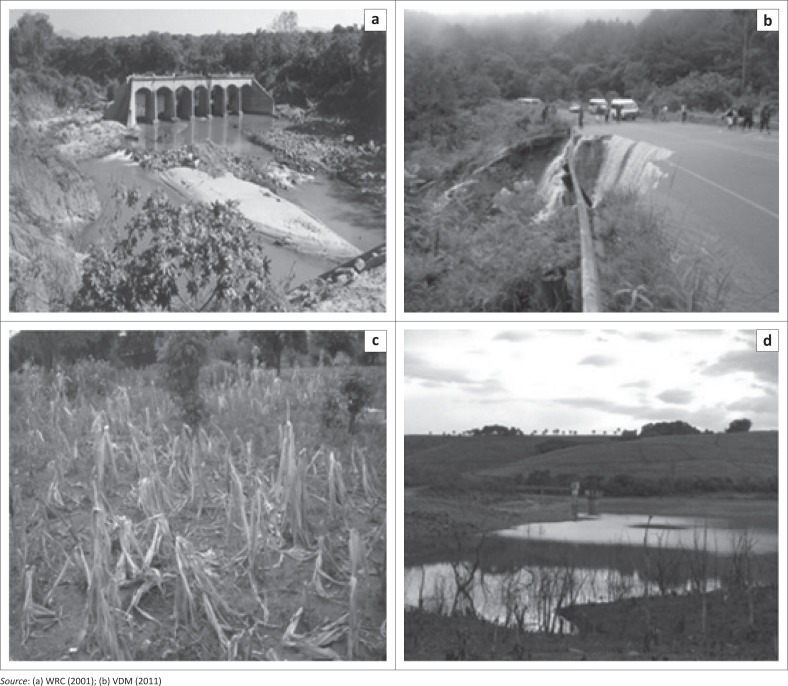
Examples of the impacts of hydrological hazards in Vhembe District Municipality: (a) Latonyanda Bridge collapse (1999–00 flood); (b) Thathe Vondo Bridge collapse 2011–12 flood); (c) Crope failure at Mhinge Village (2014–15 grought); (d) Reduced water levels in Vondo Dam (08/11/2016).

Figure 1 shows a few examples of the impacts of hydrological hazards within the VDM, South Africa. Figure 1 a and b shows two bridges that collapsed as a result of 1999–2000 and 2011–2012 floods, respectively. Figure 1 c and d shows crop failures in Mhinga Village and reduced water levels at Vondo Dam, respectively, because of drought.

### Key focus

The key focus of this study was to assess hydrological hazards in VDM and quantify the risks and vulnerabilities associated with them.

### Objectives

The objectives of the study include determination of drought and flood frequencies, rainfall and streamflow trends’ analyses, and assessment of risk and vulnerabilities of communities within VDM.

### The study area

**FIGURE 2:**
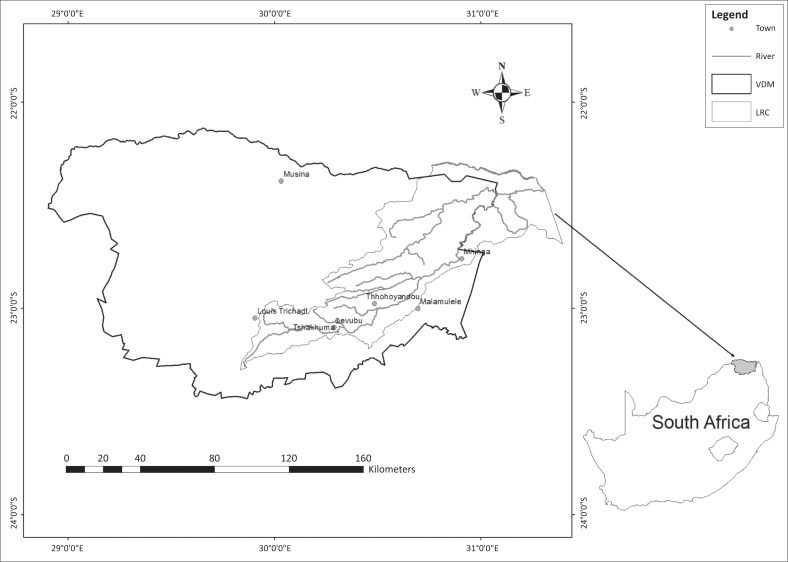
The study area.

Luvuvhu River Catchment (LRC) within VDM (Figure 2) was selected as the study area. It is situated in the northernmost part of South Africa in Limpopo province on the windward side of the Soutpansberg Mountain range, which influences the rainfall pattern in that area and also the probabilities of the occurrence of hydrological hazards and associated risks. The LRC covers an area of approximately 5941 km^2^ (DWAF 2002). Figure 2 shows the location of the study area, which lies between latitude 22^°^6’0” S and 23^°^25’0” S, and longitude 28^°^25’0’’E and 30^°^55’0’’E.

The quantity as well as spatial–temporal distribution of precipitation within VDM is highly variable (Odiyo, Makungo & Nkuna 2015). High rainfall is usually experienced in Tshakhuma and Levubu areas, while Mhinga Village receives very little rainfall. The LRC was selected because it is the economic hub of VDM with major commercial farms located in the Levubu Valley. There have also been a number of destructive floods and droughts with serious consequences on infrastructure and communities. Regular occurrences of hydrological hazards negatively impact agricultural production and rural communities’ livelihoods. The LRC is generally characterised by limited high potential agricultural soil. Soils acquired in LRC showed sandy, clay and loamy textures.

## Methodology

### Data sources and acquisition

Daily rainfall data from 12 rainfall stations for 1959–1960 to 2007–2008 were obtained from South African Weather Service (SAWS) and Department of Water and Sanitation (DWS), while daily streamflow data for six streamflow gauging stations for 1959–1960 to 2013–2014 were obtained from DWS.

**FIGURE 3:**
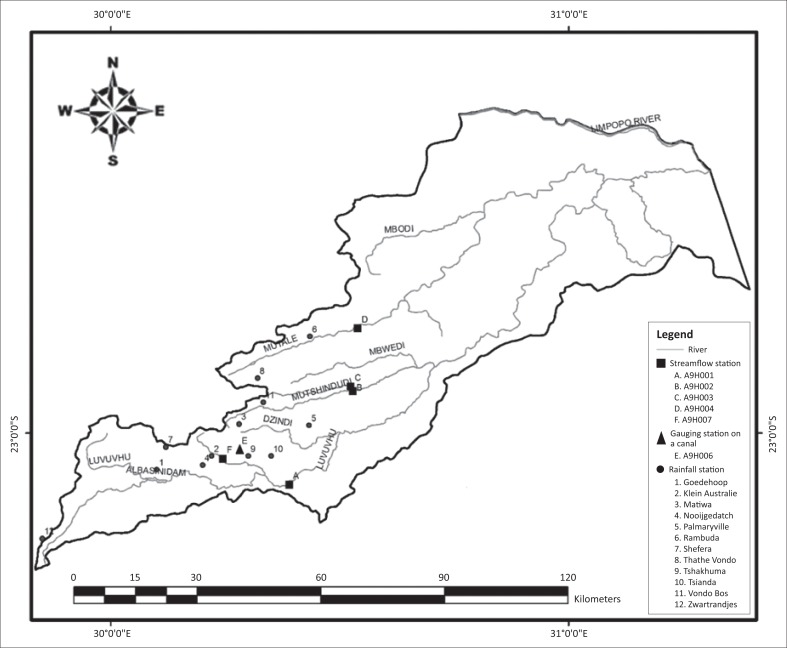
Locations of rainfall and streamflow stations.

The study considered rainfall and streamflow stations in the upper and middle reaches of the catchment. Stations in the lower reaches of the catchment were not suitable for the study because they did not have adequate data to cover the minimum required period (30 years) considered in this study because of gaps and/or short records. This limited the number of stations used in the study. World Meteorological Organization (WMO) recommended 30 years as ideal for studying long-term climate change and trends’ analysis. MacKellar, New and Jack (2014) used 50-years-long data to model rainfall and temperature trends in South Africa. Study periods adopted for this study were 33–49 and 39–55 years for rainfall and streamflow, respectively, because both rainfall and streamflow data from selected stations varied in the length of period of data availability. Figure 3 shows locations of rainfall and streamflow stations in LRC.

### Determination of flood frequency

Flood peaks corresponding to return periods (Tr_s_) of 2, 5, 10, 50, 100 and 200 years were estimated using Log-Pearson Type III (LP3) distribution. Log-Pearson Type III is a member of the family of Pearson Type 3 distributions (Millington et al. 2011) defined by the mean, standard deviation and skew coefficient of the logarithms of stream discharge (Oberg & Mades 1987). Log-Pearson Type III is used for estimating recurrence interval of flood events. Streamflow data were used to estimate Tr_s_, followed by extrapolating 100 and 200 years’ flood events from the plot. The details of the six streamflow stations used in this study are given in Table 1. Owemoje and Owemooje (2011) suggested that in predicting maximum floods with Tr_s_ of 25-year and 50-year periods, LP3 should be used with Weibull plotting position. Weibull plotting position refers to the probability value assigned to each streamflow event to be plotted; thus it uses the relationship between streamflow against corresponding probability of exceedance. Log-Pearson Type III has been widely used in South Africa to estimate Tr_s_ of floods (Alexander 2001; Singo et al. 2012). Shorter Tr_s_ were used to assess the effects of floods on rural communities, particularly on food production and daily livelihood.

**TABLE 1:** Selected rainfall and streamflow stations and their geographic location reflecting years of data coverage.

Station number	Station type	Station name	Station location	Data span	Data length
A9H001	Streamflow	Luvuvhu River @ Weltevreden	Lat - 23.11242, Lon - 30.38949	1960–2005	46
A9H002	Streamflow	Mutshindudi River @ Chibase	Lat - 22.90799, Lon - 30.52810	1960–1999	40
A9H003	Streamflow	Tshinane River @ Chibase	Lat - 22.89814, Lon - 30.52378	1959–2013	55
A9H004	Streamflow	Mutale River @ Tengwe	Lat - 22.77105, Lon - 30.53894	1960–2004	45
A9H006	Streamflow	Livhungwa River @ Barotta	Lat - 23.03572, Lon - 30.27748	1963–2014	52
A9H007	Streamflow	Latonanda River @ Levubu Settlement	Lat - 23.05575, Lon - 30.24476	1961–1999	39
0723155 X	Rainfall	Goedehoop	Lat - 23.08, Lon - 30.10	1959–1992	34
0723363 X	Rainfall	Klein Australie	Lat - 23.0500, Lon - 30.22	1959–2007	49
0766509 9	Rainfall	Matiwa	Lat - 22.9800, Lon - 30.28	1959–2008	50
0723603 0	Rainfall	Tsianda	Lat - 23.0550, Lon - 30.350	1959–2008	50
0723334 X	Rainfall	Nooitgedatch	Lat - 23.0700, Lon - 30.20	1959–2008	50
0723182	Rainfall	Shefera	Lat - 23.0300, Lon - 30.1200	1959–2008	50
0766827 4	Rainfall	Rambuda	Lat - 22.7889, Lon - 30.4347	1959–2008	50
0766596 9	Rainfall	Vondo Bos	Lat - 23.9330, Lon - 30.333	1963–2008	46
0766779 6	Rainfall	Palmaryville	Lat - 22.9830, Lon - 30.433	1959–2008	50
0723513X	Rainfall	Tshakhuma	Lat - 23.0500, Lon - 30.300	1959–2008	50
0722614 3	Rainfall	Zwartranjies	Lat - 23.2300, Lon - 29.85	1959–2008	50
0766563 1	Rainfall	Thathe Vondo	Lat - 22.8800, Lon - 30.32	1963–2000	38

To assess how floods affect built-up areas, the degree of protection was considered for floods with Tr of 100 or 200 years. Each computed flood magnitude was determined at 95% confidence interval, which contained the true flood magnitude for a particular exceedance probability. Annual daily maximum (ADM) flood discharge series were extracted for each hydrological year (starting from October of one year to September of the following year). Estimated discharge values *X_T_* for a given period were evaluated using the logarithm of the design flood given as:

$$X_{T}=\log Q_{T}=X_{av}+K\sigma_{X}$$ [Eqn 1]

where *Q*_*T*_ is the discharge for estimated Tr, *K* is the probability factor based on *n*-years recurrence interval, *X*_*av*_ is the mean of the logarithms of annual peak flows (*X_T_*) and *σ*_*x*_ is the standard deviation regarding the mean of the logarithm of ADM. Skewness coefficient *G* (Eqn. 2) was computed as an important hydrological characteristic that gives a measure of sampling distribution shape.

$$G=\frac{n\sum {\left(X-\bar{X}\right)}^{3}}{(n-1)(n-2)S^{3}}$$ [Eqn 2]

*X* is the logarithm of annual peak flow, $\bar{X}$ is sample mean, *n* is length of data set and *S* is sample standard deviation. The probability of occurrence of flood peaks corresponding to Tr_s_ of 2, 5, 10, 50, 100 and 200 years for each station was computed using Eqn 3. The regression line from the probability plot was extended to cover Tr of 50, 100 and 200 years.

$$P=\frac{1}{T_{r}}$$ [Eqn 3]

*P* is the probability of occurrence.

### Standardised Precipitation Index

Standardised Precipitation Index (SPI) (McKee, Doesken & Kleist 1993) uses a standardisation procedure that transforms rainfall data to derive standardised anomalies. Rainfall data for 12 stations covering 34–50 years (Table 1) were used in the analysis. Standardisation procedure aids in discerning normal and typical values and is symmetrical for the occurrence of wet and dry events (Sutton and Kempi 1993). The data were standardised using the following equation, as defined by Goddard and Melville (1996):

$$Z=\frac{x_{i}-\bar{x}}{s}$$ [Eqn 4]

where $\bar{x}$ is the sample mean, *Z* is the normalised standardised departure, *x_i_* is the six-months mean value and *s* is the sample standard deviation.

Six months SPI (SPI-6) was selected for drought frequency analysis. Six months SPI at the end of March gives a very good indication of the amount of precipitation that has fallen during the wet period (WMO 2012). The wet period in the study area is from October to March, and thus SPI-6 has the ability to show deficiency in rainfall, which is an indicator of drought. Standardised Precipitation Index is particularly suited to compare drought conditions among different time periods and regions with different climates (Cacciamani et al. 2007). Standardised Precipitation Index categories (Table 2) defined by WMO (2012) were used to classify drought years as they included Tr_s_ for drought events within each category.

**TABLE 2:** Probabilities of recurrence for different SPI values and categories.

SPI	Category	Tr
0 to −0.99	Mild dryness	3
−1.00 to −1.49	Moderate dryness	10
−1.5 to −1.99	Severe dryness	20
< −2.0	Extreme dryness	50

*Source*: Adapted from WMO (2012)

SPI, Standardised Precipitation Index.

### Rainfall and streamflow trend detection

In this study, Mann–Kendall (non-parametric) trend test (Kendall 1975; Mann 1945) was used to detect trends and their significance for ADM rainfall and streamflow. Mann–Kendall trend test (Kendall & Gibson 1990; Mann 1945) has been widely applied and has the advantage that the power and significance are not affected by actual distribution unlike the parametric distributions. This method has been applied widely in hydro-meteorological trend analysis studies (Burn & Hesch 2007; Hamed 2008; Hirsh, Slack & Smith 1982; Longobardi & Villani 2009; Nenwiini & Kabanda 2013; Nury, Koch & Alam 2014; Odiyo et al. 2015).

Paired two-tailed *t*-tests were used to verify significant or non-significant differences between ADM rainfall and streamflow trends. Trends were analysed using a 95% confidence level (i.e. a significance level [alpha] of 0.05). The null hypothesis (H_o_), which suggests there is no trend, was tested against the alternative hypothesis (H_1_), which suggests there is a trend. Mann–Kendall trend test analyses the sign of the difference between later and earlier measured data values (Nury et al. 2014). Each later value is compared to all values measured earlier, resulting in a total number of observations. Initial value of Mann–Kendall statistic, *S*, is zero, and this indicates that there is no trend in data series (Hirsh et al. 1982). If *S* is a large positive number, later values tend to be larger than earlier values and an upward trend is indicated. When *S* is a large negative number, later values tend to be smaller than earlier values and a downward trend is indicated.

### Risks and vulnerabilities

In this study, risk was adopted from Wilhite, Sivakumar and Pulwarty (2014) using both exposure of a location to drought or flood hazard and vulnerability of that location to periods of drought-induced water shortages or water surplus as a result of floods. The likelihood and consequence categories for the risk matrix were determined for each flood event based on a methodology by Ayyub (2003). The corresponding consequence level (CL) of each flood event was multiplied by its likelihood (LL) in order to find out the risk rating (RR) for each impact using Eqn. 5:

RR=CL×LL [Eqn 5]

CL and LL were obtained based on Tr and probability of occurrence of a flood event. In this case, CL and LL categories were assigned corresponding to each Tr and probability. These were later described in terms of their frequency of occurrence and their likely impact. For drought risk analysis, LL was determined from likelihood table based on computed probabilities of occurrence for each drought category within each station following UV and NDGDM (2013). Standardised consequence table from UV and NDGDM (2013) was used to determine CL. Qualitative risk matrix combined LL and CL to determine the risk level. In this study, vulnerability was based on how the impact affects rural communities, particularly on food production and daily livelihoods. Risk rating shows the vulnerability of rural communities to hydrological hazards.

## Results and discussions

### Flood frequency analysis

**FIGURE 4:**
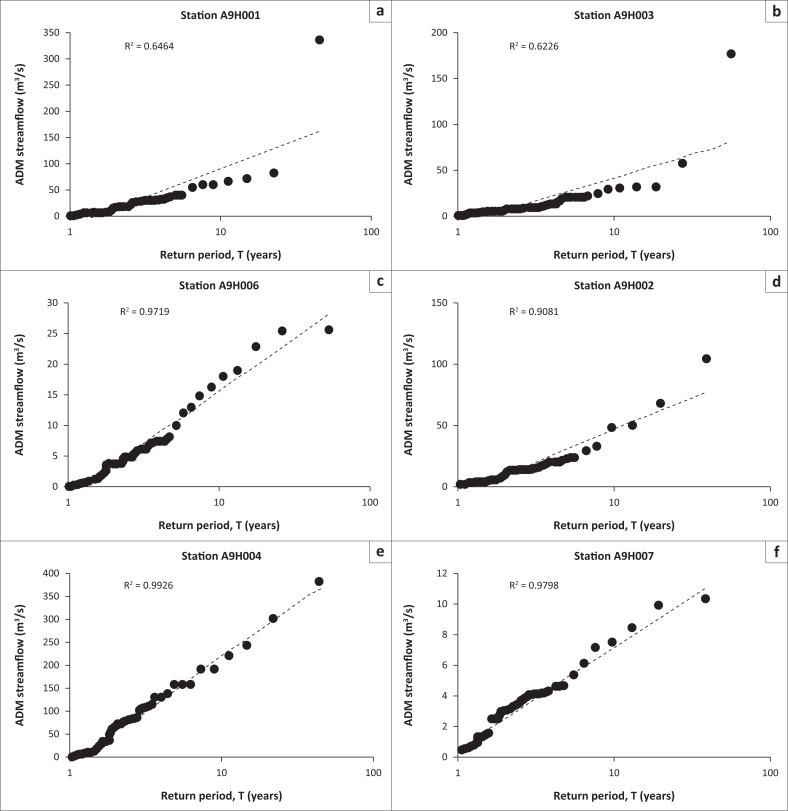
Annual daily maximum streamflow versus Tr plot for selected stations in the study area.

Figure 4 depicts Tr plot of ADM streamflow for stations A9H001, A9H002, A9H003, A9H004, A9H006 and A9H007 in the study area, with each station’s coefficient of determination (*R*^2^). *R*^2^ describes the degree of collinearity between two variables and ranges from zero to one, with higher values indicating less error variance, and typically, values greater than 0.5 are considered acceptable (Santhi et al. 2001; Van Liew, Arnold & Garbrecht 2003). There is a linear relationship between ADM streamflow and Tr with *R*^2^ of stations A9H001 and A9H003 being 0.6464 and 0.6226, respectively. The latter stations showed the lowest linear relationships of the six stations. Stations A9H002, A9H004, A9H006 and A9H007 have *R*^2^ of above 0.9. Although stations A9H001 and A9H003 reported lower *R*^2^, it is still greater than 0.5, which is indicative of a linear relationship. These results indicate that linear relationship between Tr and ADM streamflow from stations A9H001 and A9H003 is less significant than that of stations A9H002, A9H004, A9H006 and A9H007.

Flood peaks corresponding to Tr of 2, 5, 10, 25, 50, 100 and 200 years were estimated for flood prevention and protection from risks in the catchment at different probabilities of exceedance. Log-Pearson Type III estimated discharges are shown in Table 3.

**TABLE 3:** Tr and probability of annual daily maximum streamflow.

Tr	A9H001		A9H003		A9H006		A9H002		A9H007		A9H004	
	P (%)	Q (m^3^/s)	P (%)	Q (m^3^/s)	P (%)	Q (m^3^/s)	P (%)	Q (m3/s)	P (%)	Q (m^3^/s)	P (%)	Q (m^3^/s)
2	1	14.01	1	6.06	1	2.97	1	9.05	1	3	1	67.77
5	0.999	39.45	0.999	20.07	0.999	9.1	0.999	21.14	0.999	4.69	0.999	155.14
10	0.994	61.99	0.994	28.84	0.994	18.68	0.994	48.18	0.994	7.46	0.994	206.59
25	0.923	101.53	0.923	50.3	0.923	24.85	0.923	76.55	0.923	9.99	0.923	310.42
50	0.635	169.00	0.635	80.00	0.635	28.00	0.635	82.00	0.635	11.80	0.635	340.00
100	0.394	197.00	0.394	100.00	0.394	32.00	0.394	98.00	0.394	13.80	0.394	450.00
200	0.221	230.00	0.221	110.00	0.221	40.00	0.221	113.00	0.221	16.00	0.221	520.00

The lowest Tr corresponded to high probability of occurrence (see Eqn. 3) and low estimated streamflow for each station. A two-year Tr with a 100% probability of occurrence corresponded to 14.01, 6.06, 2.97, 9.05, 3.00 and 67.77 m^3^/s for stations A9H001, A9H003, A9H006, A9H002, A9H007 and A9H004, respectively. Because of variation in data length, streamflows corresponding to Tr of 50, 100 and 200 years were extrapolated for stations A9H001, A9H002, A9H004 and A9H007, while for station A9H003 and A9H006 extrapolation was performed for only streamflow corresponding to Tr of 100 and 200 years. Extrapolated streamflow showed that flood magnitude increases with a lower probability of occurrence and a high Tr. –Two hundred years Tr at 22.1% probability of occurrence corresponded to 230, 110, 40, 113, 16 and 520 m^3^/s for stations A9H001, A9H003, A9H006, A9H002, A9H007 and A9H004, respectively. Results of this study, therefore, indicate that a low magnitude flood with a low Tr has the highest probability of occurrence, while a high magnitude flood with a high Tr has the lowest probability of occurrence and is likely to result in the largest damage in the study area. Karlsson and Haimes (1988) indicated that a low probability expectation is a measure of the average largest damage, given the events of an extreme nature. Therefore, the results of this study follow the concept of ‘high probability/low damage’ and ‘low probability/high damage’ as explained in Merz et al. (2009).

### Drought frequency analysis

**FIGURE 5:**
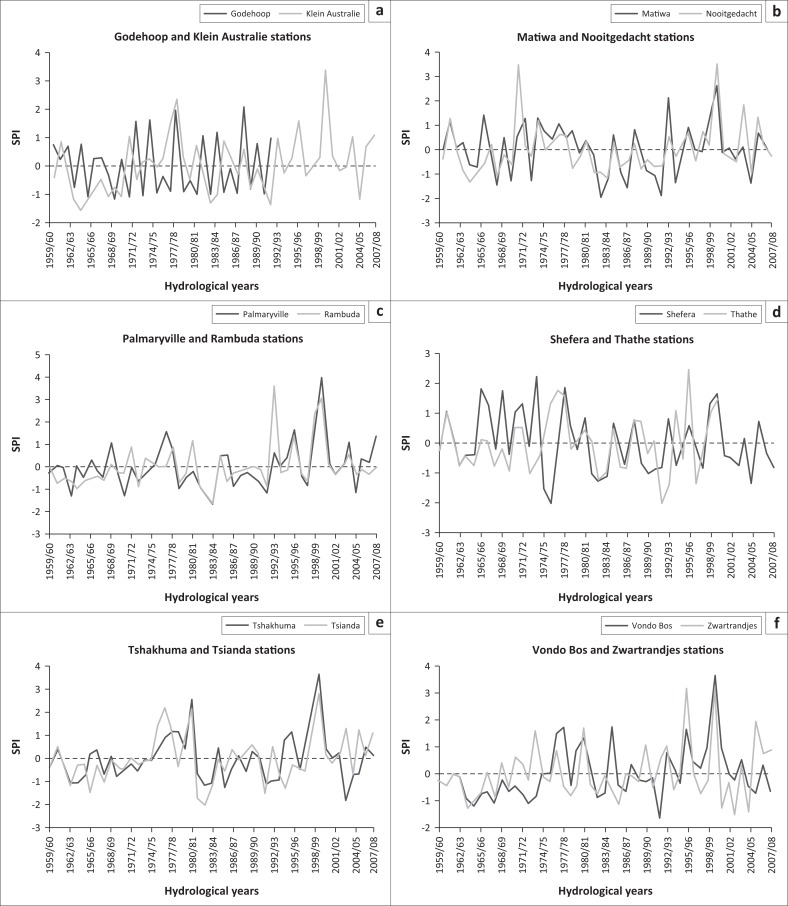
Standardised Precipitation Index for stations in the study area.

Figure 5 displays fluctuations of wet and dry years, which show the variable nature of rainfall in the study area. Mild dryness condition dominated the dry years in all stations with probabilities of occurrence ranging from 22.4% to 59.2% (Table 4). Mild dryness condition has a Tr of three years, showing that the prevalence of occurrence of mild dryness is high in the study area. Communities within the vicinity of these stations are therefore regularly affected by droughts. Moderate dryness condition had a probability of occurrence from 4.1% to 18.4% with a Tr of 10 years. The latter, just like the mild dryness category, has a high prevalence in the study area. Severe dryness had a probability of occurrence of 2% in most of the stations, while extreme dryness condition had a probability of occurrence of 2% in Rambuda and Thathe. Six stations (Palmaryville, Rambuda, Shefera, Tsianda, Vondo Bos and Zwartrandjes) each had a probability of occurrence of dry years exceeding 50%, with Palmaryville and Rambuda having the highest percentages of probability of occurrence of dry years of 63.2% and 65.3%, respectively (Table 4). This shows the prevalence of drought conditions in the areas within the vicinity of these stations. Tsianda, Palmaryville and Rambuda also had more than 50% probabilities of occurrence of mild dryness.

**TABLE 4:** Tr and probabilities of occurrence within each SPI category.

Category	Tr	GP	KA	MA	NT	PE	RA	SA	TT	TS	TA	VB	ZS
Mild dryness	3	28.6	28.6	22.4	22.4	51.0	59.2	42.9	24.5	36.7	51.0	44.9	36.7
Moderate dryness	10	8.2	16.3	18.4	6.1	10.2	4.1	8.2	12.2	6.1	6.1	10.2	12.2
Severe dryness	20	-	2.0	2.0	0.0	2.0	0.0	0.0	0.0	2.0	2.0	2.0	2.1
Extreme dryness	50	0.0	0.0	0.0	0.0	0.0	2.0	0.0	2.0	0.0	0.0	0.0	0.0
**Total**		**36.7**	**46.9**	**42.9**	**28.5**	**63.2**	**65.3**	**51.1**	**38.7**	**44.8**	**59.1**	**57.1**	**51.0**

GP, Godehoop; KA, Klein Australie; MA, Matiwa; NT, Nooitgedacht; PE, Palmaryville; RA, Rambuda; SA, Shefera; TT, Thathe; TS, Tshakhuma; TA, Tsianda; VB, Vondo Bos; ZS, Zwartrandjes; SPI, Standardised Precipitation Index.

Seven stations (Palmaryville, Matiwa, Tsianda, Rambuda, Shefera, Thathe and Zwartrandjes) displayed the 1997–1998 (Figure 5) drought that was recorded in the north-eastern interior region of South Africa during El Nino in a study by Rouault and Richard (2003). Lindesay and Vogel (1990) and Edossa, Woyessa and Welderufael (2014) have shown that El Nino is usually associated with drought condition in South Africa. The years 1982–1983 and 1991–1992 in moderate and extreme dryness categories were classified as the two strongest droughts within the last two decades, by Rouault and Richard (2003). These droughts are clearly shown in the selected stations in this study (Figure 5). Other notable drought years as depicted by the SPI plot of the stations in the LRC include but are not limited to 1962–1963, 1964–1965, 1972–1973, 1986–1987, 1993–1994, 2001–2002 and 2004–2005.

### Rainfall and streamflow trend analysis

Mann–Kendall trends test results for rainfall and streamflow are presented in Table 5 and Table 6, respectively. The stations with significant positive or negative trends are reported at a confidence interval of 95%. Longobardi and Villani (2009) used non-parametric trend test at confidence levels of 99%, 95% and 90% while assessing trends of annual and seasonal rainfall time series in the Mediterranean area. The latter study successfully detected trends at 99%, 95% and 90% confidence levels and reported predominant negative trends at both annual and seasonal scales with exception of summer months when the trend appeared to be positive. Karmeshu (2012) tested the null hypothesis at 95% confidence level for temperature and precipitation data in nine states in the USA. Results of Karmeshu (2012) found significantly increasing trends for both precipitation and temperature, for seven of the eight states.

**TABLE 5:** Mann–Kendall trends for rainfall stations.

Station	*S*	*p* (two tailed test	Alpha	Test interpretation	Trend
Shefera	−87.000	0.445	0.05	Accept H_o_	Downward
Tsianda	47.000	0.692	0.05	Accept H_o_	Upward
Tshakhuma	−88.000	0.453	0.05	Accept H_o_	Downward
Palmaryville	86.000	0.000	0.05	Reject H_o_	Upward
Entabeni-Bos	85.000	0.469	0.05	Accept H_o_	Upward
Klein-Australie	276.000	0.017	0.05	Reject H_o_	Upward
Nooitgedacht	89.000	0.448	0.05	Accept H_o_	Upward
Matiwa	−31.000	0.790	0.05	Accept H_o_	Downward
Rambuda	288.000	0.011	0.05	Reject H_o_	Upward
Vondo Bos	240.000	0.016	0.05	Reject H_o_	Upward
Thathe	98.000	0.204	0.05	Accept H_o_	Upward
Zwartrandjes	62.000	0.588	0.05	Accept H_o_	Upward

**TABLE 6:** Mann–Kendall trends for selected streamflow stations.

Station	*S*	*p* (two tailed test	Alpha	Test interpretation	Trend	Significant
A9H001	30.00	0.778	0.05	Accept H_o_	Upward	No
A9H003	386.00	0.004	0.05	Reject H_o_	Upward	Yes
A9H006	204.00	0.109	0.05	Accept H_o_	Upward	No
A9H002	−740.00	<0.0001	0.05	Reject H_o_	Downward	Yes
A9H004	−902.00	<0.0001	0.05	Reject H_o_	Downward	Yes
A9H007	−700.00	<0.0001	0.05	Reject H_o_	Downward	Yes

About 25% of rainfall stations depicted downward trends, while 75% showed positive upward trends for the rainfall time series. Of the nine stations that exhibited an upward trend, four stations showed that the trend was statistically significant, while Tsianda, Entabeni-Bos, Nooitgedatch, Thathe and Zwartrandjes stations had insignificant positive trends. All stations that exhibited downward trends reported insignificant trends. About 16.7% of streamflow stations depicted downward trends, while 83.3% depicted upward trends. Upward trends for streamflow were statistically significant for station A9H003 and insignificant for stations A9H001 and A9H006. Stations A9H002, A9H004 and A9H007 all exhibited significantly downward trends.

The results on trends analyses obtained in this study are comparable with those obtained by Odiyo et al. (2015) with some stations having the same trend characteristic. For example, Odiyo et al. (2015) found that Zwartrandjes rainfall station had an insignificant upward trend, while the streamflow station A9H006 also reported the same results. The latter comparability is not the case for the rest of the stations, this may be because Odiyo et al. (2015) used annual total time series, while this study used ADM time series for both rainfall and streamflow.

### Risk and vulnerabilities

Table 7 shows quantified flood risk in the study area, including likelihood and consequence of the flood event. The likelihood of flood occurrence ranged from the probability of 0.22 to 1.00. Because of the latter, the identified risks associated with floods had probabilities of more than 0.22. This means that flood risks of category A with LL of six were likely to occur.

**TABLE 7:** Flood risk in the study area.

Station number	Tr	P	Likelihood			Consequence			RR	Station number	Tr	P	Likelihood			Consequence			RR
			C	D	L	C	L	D					C	D	L	C	L	D	
A9H001	2	1	A	Likely	6	A	1	None	6	A9H002	2	1	A	Likely	6	A	1	None	6
	5	0.999	A	Likely	6	B	2	Minor	12		5	0.999	A	Likely	6	A	1	None	6
	10	0.994	A	Likely	6	D	4	Serious	24		10	0.994	A	Likely	6	C	3	Significant	18
	25	0.923	A	Likely	6	E	5	Major	30		25	0.923	A	Likely	6	C	3	Significant	18
	50	0.635	A	Likely	6	F	6	Catastrophic	36		50	0.635	A	Likely	6	E	5	Major	30
	100	0.394	A	Likely	6	F	6	Catastrophic	36		100	0.394	A	Likely	6	E	5	Major	30
	200	0.221	A	Likely	6	F	6	Catastrophic	36		200	0.221	A	Likely	6	F	6	Catastrophic	36
A9H003	2	1	A	Likely	6	A	1	None	6	A9H007	2	1	A	Likely	6	A	1	None	6
	5	0.999	A	Likely	6	A	1	None	6		5	0.999	A	Likely	6	A	1	None	6
	10	0.994	A	Likely	6	B	2	Minor	12		10	0.994	A	Likely	6	A	1	None	6
	25	0.923	A	Likely	6	C	3	Significant	18		25	0.923	A	Likely	6	B	2	Minor	12
	50	0.635	A	Likely	6	C	3	Significant	18		50	0.635	A	Likely	6	C	3	Significant	18
	100	0.394	A	Likely	6	F	6	Catastrophic	36		100	0.394	A	Likely	6	F	6	Catastrophic	36
	200	0.221	A	Likely	6	F	6	Catastrophic	36		200	0.221	A	Likely	6	F	6	Catastrophic	36
A9H006	2	1	A	Likely	6	A	1	None	6	A9H004	2	1	A	Likely	6	B	2	Minor	12
	5	0.999	A	Likely	6	A	1	None	6		5	0.999	A	Likely	6	B	2	Minor	12
	10	0.994	A	Likely	6	A	1	None	6		10	0.994	A	Likely	6	C	3	Significant	18
	25	0.923	A	Likely	6	B	2	Minor	12		25	0.923	A	Likely	6	C	3	Significant	18
	50	0.635	A	Likely	6	B	2	Minor	12		50	0.635	A	Likely	6	C	3	Significant	18
	100	0.394	A	Likely	6	C	3	Significant	18		100	0.394	A	Likely	6	E	5	Major	30
	200	0.221	A	Likely	6	F	6	Catastrophic	36		200	0.221	A	Likely	6	F	6	Catastrophic	36

C, category; D, description; L, level; RR, risk rating.

The RR for floods was assessed for Damage to Houses and Household Products (DHHP), Inundation of Roads Networks and Their Damage and Destruction (IRDD), Damage to Crops and Losses of Livestock (DCLL), Erosion of Agricultural Lands (EAL), problems in relocation of people, Spread of Diseases (SD) and Effects on Health (Hossain 2014). The latter flood impacts categories were selected based on the fact that historical floods in the study area tended to damage houses (International Federation’s Disaster Relief Emergency Fund [IFDREF] 2011), damage road networks (Figure 1a and b), affect agricultural productivity and spread of water borne diseases. The results from Table 7 showed that high T_r_ correlated with low probability and high RR.

The results showed catastrophic consequence levels for station A9H001 at 50, 100 and 200 years T_r_. Stations A9H003 and A9H007 depicted catastrophic consequence levels for both 100 and 200 years Tr. A9H002, A9H004 and A9H006 had catastrophic consequence levels for 200 years Tr. Two hundred years Tr in all stations showed a highest consequence level. Risk associated with 200 years Tr has the ability to cause major damage to houses and household products, and cause destruction to the environment. Tr of 10 years had a serious consequence level for station A9H001 and significant consequences for A9H002 and A9H004 stations. Significant consequence levels were found in stations A9H002 (10 and 25 Tr), A9H003 (25 and 50 Tr), A9H004 (10, 25 and 50 Tr), A9H006 (100 Tr) and A9H007 (50 Tr). The results therefore show that serious, significant and catastrophic DHHP, IRDD, DCLL, EAL and SD were expected to occur within the localities of these stations for floods with the above-mentioned Tr. The results of this study are comparable with that of Hossain (2014), which showed that a higher value of RR corresponds to catastrophic impacts on DHHP, IRDD, DCLL, EAL and SD.

Station A9H001 had a high RR corresponding to 50, 100 and 200 years Tr, followed by stations A9H003 and A9H007 which had a high RR for 100 and 200 years Tr. A9H002, A9H004 and A9H006 only had a high RR for 200 years Tr. The latter therefore indicates that communities in the sub-catchment A9H001 would be at risk if a 50-year flood were to occur. The overall average RR for the study area was estimated as 19. This corresponds to a CL of three and has the potential to result in significant consequences.

Table 8 shows LL, CLs and impacts of droughts with different probabilities of occurrence in the study area. The study area experiences mild dryness frequently when compared to the other three drought categories. Five rainfall stations (GP, KA, MA, NT and TV) in the mild dryness category had likely LLs (Table 3 and Table 7) that are associated with minor consequences. The rest of the stations (seven) in the mild dryness category (TS, ZS, SA, VB, PE, RA and TA) fell within almost likely likelihood with moderate consequences. Most stations dominated the moderate dryness category with major CL. This shows that minor and moderate, and major impacts associated with mild and moderate dryness, respectively, are frequent in the study area. Thus, communities are at risk and vulnerable to these hazards. Limpopo Economic Development, Environment and Tourism (LEDET) (2016) also reported that rural livelihoods in Thulamela Municipality within VDM are highly vulnerable to climate change-related impacts including drought.

**TABLE 8:** Drought probabilities, likelihood, consequence levels and impacts in the study area.

Drought category	Probability of occurrence	Stations	Likelihood	Risk level	Consequence level	Impacts^a^
Mild	20% – 30%	GP, KA, MA, NT, TT,	Likely	Medium	Minor	Isolated and temporary cases of reduced supply services, loss of employment (in the agricultural sector) and expressions of public concern
	30% – 60%	TS, ZS, SA, VB, PE, RA, TA	Almost likely	Medium	Moderate	Isolated but significant impairment of loss of ecosystems functions, ongoing reduced services to community, wide spread public protests, and mid –term failure of service delivery.
Moderate	0% – 20%	GP, NT, RA, SA, TS, TA, KA, MA, PE, TT, VB, ZS,	Likely	High	Major	Multiple loss of lives, severe impairment or loss of ecosystem functions, progressive environmental damage, multiple business failures and loss of employment, loss of public confidence in governance, reduced quality of life in community, mid to long term failure of service delivery
Severe	0% – 5%	KA, MA, PE, TS, TA, VB, ZS	Unlikely	High	Catastrophic	Widespread loss of lives, wide spread severe impairment or loss of ecosystem functions, public unrest, community unable to support itself, long-term failure of service delivery affecting all parts of community
Extreme	0% – 5%	RA, TT	Highly unlikely	Medium	Catastrophic	

a , See University of Vienna and National Directorate General for Disaster Management (2013).

GP, Godehoop; KA, Klein Australie; MA, Matiwa; NT, Nooitgedacht; PE, Palmaryville; RA, Rambuda; SA, Shefera; TT, Thathe, TS, Thakhuma; VB, Vondo Bos; ZS, Zwartrandjes; -, no years fell within SPI category.

The actual impacts of drought hazards are shown in Table 8 and are related to reduced supply or delivery services, loss of employment (in the agricultural sector), loss of ecosystems functions and public reactions. Table 8 also shows that the lower the probability of occurrence, the more severe the drought will be. Although these events have lower probabilities of occurrence, their impacts are catastrophic and persist for a long period after the occurrence of the events. Thus, it would take long for communities to recover from such events. Unlike flood, drought will not cause destruction to infrastructure and household products. Rather, drought will affect water-depended services, including agriculture, industries and household water. The effects of drought result in high food prices, contribute to food insecurity and a negative impact on the economy of an area. Maponya and Mpandeli (2012) also reported that consequences of drought in Limpopo province include reduced grazing and water for livestock and irrigation and hence resulting in food scarcity. The latter study noted that farmers also sell their livestock to cope with reduced availability and higher prices of livestock feed during drought.

Catastrophic CLs were found in stations KA, MA, PE, TS, TA, VB and ZS (20 and 50 Tr), which are unlikely to occur or have very low chances of occurrence (2% probability) (Tables 4 and 8). Stations RA and TV had catastrophic CLs that are highly unlikely to occur within 20 and 50 years Tr. The latter CL can lead to severe water shortages that can result in imposition of water rationing. Major, moderate and minor CLs affect water resources availability at a rate less than the catastrophic CLs. Minor CL has the least impact on both the environment and communities.

The risk levels for drought categories vary from medium to high (Table 8). Risk level categories determine whether the risk treatment measures are required. Thus, medium to high risks call for the implementation of drought mitigation measures in the study area. Mpandeli (2014) reported that small-scale farmers in Vhembe District use different drought adaptive strategies, including drought-resistant varieties, crop diversification, plant crops that require less water, use local climate indicators to monitor climate risk, adjust fertiliser input and apply rainwater harvesting techniques. Drought relief programmes are implemented to combat water shortages in South Africa, including the study area, during drought periods. This includes drilling a number of emergency boreholes for groundwater supply, and rehabilitation and refurbishment of existing boreholes. These programmes were implemented during the 1992–1993 and 2015–2016 (Department of Water and Sanitation 2016) droughts, for example.

## Conclusions

This study determined the hydrological hazards as well as the risks and vulnerabilities associated with these hazards. Annual daily maximum streamflow series was successfully fitted to an LP3 distribution for a period between 39 and 55 years. Mild dryness condition dominated the dry years in all stations with a range of 22.4% to 59.2% of the years falling within this category. Rainfall and streamflow showed both negative and positive trends, thus displaying their highly variable nature in the study area. The study generally shows that flood events are mostly associated with serious, significant and catastrophic CLs in the study area, making it highly prone to flood impacts such as loss of lives and damages to infrastructure. Additionally, CLs of different flood magnitudes have been determined in the VDM that had not been done before. The study also showed that communities in the study area are vulnerable to mild and moderate dryness conditions, which are associated with minor and moderate, and major impacts, respectively. Thus, there is high risk and vulnerability of communities to these hazards. The findings will aid in strategic mitigation of hydrological hazards in the study area.
